# Root-associated microbiomes of wheat under the combined effect of plant development and nitrogen fertilization

**DOI:** 10.1186/s40168-019-0750-2

**Published:** 2019-10-22

**Authors:** Shuaimin Chen, Tatoba R. Waghmode, Ruibo Sun, Eiko E. Kuramae, Chunsheng Hu, Binbin Liu

**Affiliations:** 1 Key Laboratory of Agricultural Water Resources, Hebei Key Laboratory of Soil Ecology, Center for Agricultural Resources Research, Institute of Genetics and Developmental Biology, Chinese Academy of Sciences, 286 Huaizhong Road, Shijiazhuang, 050021 China; 2 University of Chinese Academy of Sciences, Beijing, 100039 China; 3 Department of Microbial Ecology, Netherlands Institute of Ecology (NIOO-KNAW), 6708 Wageningen, PB Netherlands

**Keywords:** Root-associated microbiomes, Root exudate, Organic acid, Nitrogen fertilization, Plant growth stage

## Abstract

**Background:**

Plant roots assemble microbial communities both inside the roots and in the rhizosphere, and these root-associated microbiomes play pivotal roles in plant nutrition and productivity. Although it is known that increased synthetic fertilizer input in Chinese farmlands over the past 50 years has resulted in not only increased yields but also environmental problems, we lack a comprehensive understanding of how crops under elevated nutrient input shape root-associated microbial communities, especially through adjusting the quantities and compositions of root metabolites and exudates.

**Methods:**

The compositions of bacterial and fungal communities from the roots and rhizosphere of wheat (*Triticum aestivum L.*) under four levels of long-term inorganic nitrogen (N) fertilization were characterized at the tillering, jointing and ripening stages. The root-released organic carbon (ROC), organic acids in the root exudates and soil organic carbon (SOC) and soil active carbon (SAC) in the rhizosphere were quantified.

**Results:**

ROC levels varied dramatically across wheat growth stages and correlated more with the bacterial community than with the fungal community. Rhizosphere SOC and SAC levels were elevated by long-term N fertilization but varied only slightly across growth stages. Variation in the microbial community structure across plant growth stages showed a decreasing trend with N fertilization level in the rhizosphere. In addition, more bacterial and fungal genera were significantly correlated in the jointing and ripening stages than in the tillering stage in the root samples. A number of bacterial genera that shifted in response to N fertilization, including *Arthrobacter*, *Bacillus* and *Devosia*, correlated significantly with acetic acid, oxalic acid, succinic acid and tartaric acid levels.

**Conclusions:**

Our results indicate that both plant growth status and N input drive changes in the microbial community structure in the root zone of wheat. Plant growth stage demostrated a stronger influence on bacterial than on fungal community composition. A number of bacterial genera that have been described as plant growth-promoting rhizobacteria (PGPR) responded positively to N fertilization, and their abundance correlated significantly with the organic acid level, suggesting that the secretion of organic acids may be a strategy developed by plants to recruit beneficial microbes in the root zone to cope with high N input. These results provide novel insight into the associations among increased N input, altered carbon availability, and shifts in microbial communities in the plant roots and rhizosphere of intensive agricultural ecosystems.

## Background

The plant microbiome equips the host plant with additional gene pools and is therefore often referred to as the second plant genome or extended genome [1–3]. Notably, plant root-associated microbiomes have attracted unprecedented attention in recent years owing to their essential roles in host nutrition, development and immunity [4]. A recent study showed that plant roots assemble microbial communities in the root zone from surrounding soil [4]. The microbiota in these compartments can be beneficial or harmful to the host plant, and a shift in this balance might substantially affect crop production in agricultural ecosystems. Therefore, understanding how root-associated microbial communities respond to soil management practices and plant physiological status is of great agronomic interest.

China is the largest consumer of chemical nitrogen (N) fertilizer in the world and applies more than 30% of global fertilizers to only approximately 9% of global cropland (FAOSTAT, www.fao.org). The high rate of N loss and low use efficiency are major problems in most agricultural areas in China [5]. For instance, in the North China Plain (NCP), one of the largest crop production areas in China, nearly 300 kg N ha^− 1^ of N fertilizer is used in one wheat growing season, accounting for an estimated overuse of more than 30% [6]. The overuse of N fertilizer has resulted in a series of environmental issues, such as groundwater nitrate contamination [7], increased greenhouse gas emissions [8] and soil acidification [9]. In particular, it is estimated that N fertilizer-induced N_2_O emissions were 460 Gg N yr^− 1^ higher in 2005 than in 1980 [8]. The available N that can be assimilated by plants is strongly dependent on root-associated microbial guilds [10]. However, it has not been investigated how root zone microbiomes respond to changes in N availability and the consequent changes in plant root exudates in areas receiving N applications as high as those in the NCP.

Carbon levels in root exudates and rhizosphere soil are important factors influencing the microbial communities related to plant N uptake. For example, arbuscular mycorrhizal fungi were recently shown to be able to transfer N to plants, and this fungal symbiont-mediated N uptake was stimulated by carbon supplied from the host plant [11]. The labile organic carbon released from the plant root can stimulate or suppress the mineralization of soil organic matter, which is an important aspect of plant-soil interactions in the rhizosphere and termed the rhizosphere priming effect [12, 13]. Growing evidence suggests that rhizosphere priming is an important strategy by which plants retrieve organic N [10], and in forest ecosystems, the priming effect caused by elevated CO_2_ is tentatively driven by increased rhizodeposition and enhanced microbial activity [14]. Considering the importance of the carbon pool in the root zone with respect to the crop N recovery rate in agricultural ecosystems, an investigation of root-associated microbial communities under various levels of carbon availability caused by N fertilization is needed.

Root-associated microbiomes are dynamically affected by both the surrounding edaphic conditions and the host plant. Soil is considered a “microbial seed bank” [15] that provides plants with a large candidate pool of microorganisms. As a strategy to modulate their local growth conditions, plants have the capacity to change the soil environment by secreting bioactive molecules into the rhizosphere to alter edaphic conditions for soil microbiota [16]. Thus, different plant species or genotypes can recruit specific microbiota through differences in root morphologies and root exudation patterns [17, 18]. In addition, the composition of root exudates [19] and the root-associated microbial community structure are strongly affected by the plant growth stage [20]. Variations in the composition of root-associated microbiomes during plant development have been illustrated in a number of recent studies using molecular technologies [20, 21] and were suggested to be caused by changes in root exudation, though the compositions and quantities of the root exudates were not assessed in these studies.

Root exudates, which are composed of a wide spectrum of carbon-containing metabolites, such as sugars, amino acids and organic acids, represent a significant carbon cost to the host plant [22] and also act as substrates and signaling molecules for microbes, resulting in complex biogeochemical exchanges between the host plant and microbes [23, 24]. As the primary low molecular weight compounds of root exudates, organic acids have been shown to act as selective agents that shape the rhizosphere microbiome structure, stimulating the growth of specific microbial populations and/or inhibiting the development of others [25, 26]. Incubation experiments have shown that compared with carbohydrates, organic acids tend to have a greater impact on the richness and structure of the dominant taxa in the soil microbial community [26–28]. Secretion of organic acids is an important strategy used by plants to cope with a low availability of nutrients such as phosphorous and nitrogen [29, 30]. However, the effect of elevated concentrations of nutrients due to fertilizer overuse in agricultural systems on organic acid secretion and the subsequent influence on the microbial community have not been examined.

For the present study, we collected root and rhizosphere samples from wheat plants at three growth stages and grown at four levels of N fertilization. The effect of plant development and long-term N fertilization on carbon availability was assessed by determining the quantities and compositions of the root exudates and organic carbon in the rhizosphere. The bacterial and fungal communities in the rhizosphere and roots were monitored using 16S and 18S rRNA gene amplicon sequencing technology. The results of this study provide in-depth information on the root exudates, rhizosphere carbon and root-associated microbial communities across different plant growth stages and N fertilization levels.

## Results

### Carbon in the rhizosphere and root exudates

**Fig. 1:**
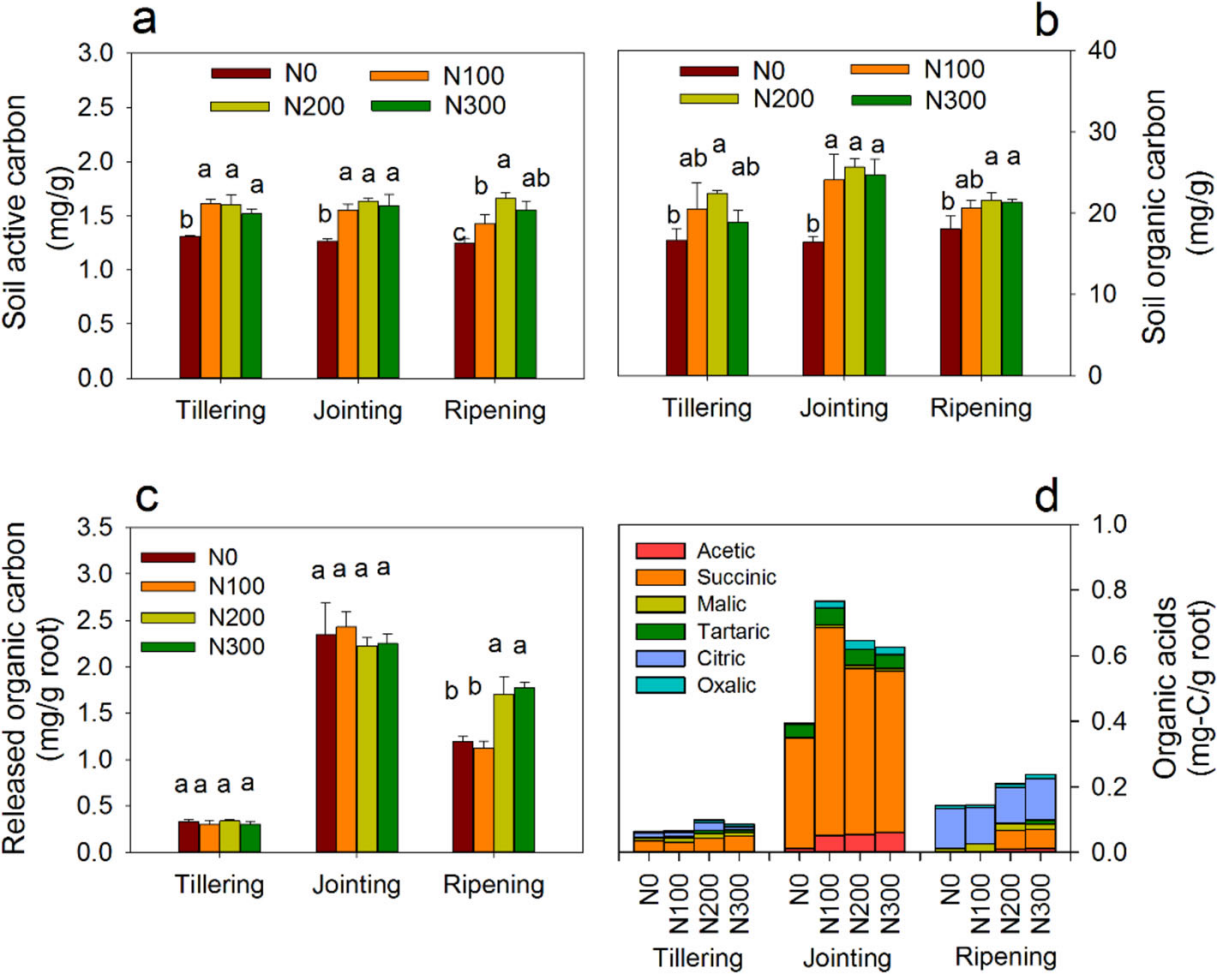
Soil active carbon (**a**), soil organic carbon (**b**), root-released organic carbon (**c**) and organic acids (**d**) in the four different N fertilization levels at three growth stages. Error bars indicate the standard deviation of three replicates. Different letters indicate significant differences (*P* < 0.05) between the N fertilization levels at each growth stage

Soil active carbon (SAC), the fraction of soil carbon that fuels the soil food web, strongly affects nutrient cycles [31]. The rhizosphere SAC was significantly lower in N0 control (without N fertilization) samples than in samples that underwent N100, N200 and N300 fertilization treatments (100, 200 and 300 kg N ha^− 1^ per wheat-growing season, respectively) at all growth stages, though no significant differences were observed among most of the fertilized samples (Fig. 1a). The levels of rhizosphere soil organic carbon (SOC) also showed a similar pattern (Fig. 1b). The root-released organic carbon (ROC) determined per unit weight of fresh root is shown in Fig. 1c. At the tillering and jointing stages, no significant difference in ROC was observed at the four N fertilization levels. At the ripening stage, the ROC in the N200 and N300 samples was significantly higher than that in the N0 and N100 samples. When the different growth stages were examined, the ROC level was higher in the jointing stage (2.23–2.43 mg/g root) than in the tillering (0.30–0.34 mg/g root) and the ripening (1.08–1.77 mg/g root) stages. Notably, the reported concentrations were normalized to root weight, and the total ROC increased with increasing level of N fertilization.

In this study, eight organic acids were assayed (acetic acid, oxalic acid, pyruvic acid, fumaric acid, succinic acid, malic acid, tartaric acid and citric acid); except for pyruvic acid and fumaric acid, all were detected in the root samples. The amount and composition of the organic acids differed according to growth stage (Fig. 1d). At the tillering stage, the sum of the organic acid concentrations was 0.06–0.10 mg C/g root with succinic acid, citric acid and malic acid dominating; these accounted for 46–62%, 17–26% and 14–20% of the assayed organic acids, respectively. At the jointing stage, the total organic acid concentration was 0.39–0.76 mg C/g root; succinic acid dominated, accounting for 82–87% of the total organic acids. The sum of the organic acid concentrations was 0.14–0.23 mg C/g root at the ripening stage, at which citric acid and malic acid were dominant and accounted for 55–92% and 7–17% of the total amount of organic acid, respectively.

### Bacterial community responses to plant development and N fertilization

**Fig. 2:**
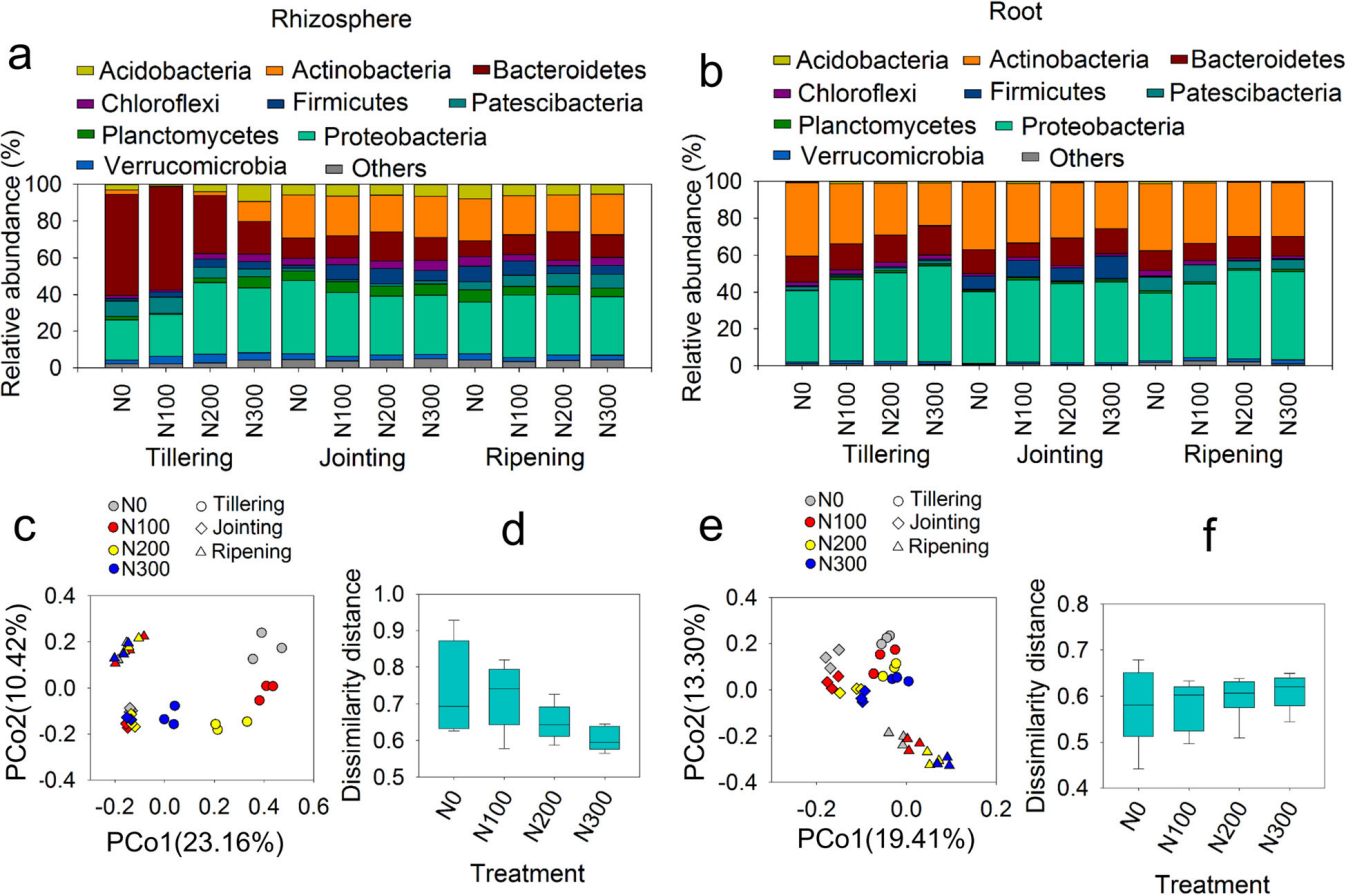
Bacterial community composition of the rhizosphere (**a**) and root (**b**) samples at the phylum level. Principal coordinate analysis (PCoA) of the bacterial communities in the rhizosphere (**c**) and root (**e**) samples. Dissimilarity distance showing the differences in microbial community structure across the growth stages in the rhizosphere (**d**) and root (**f**) samples. PCoA and dissimilarity distance were based on Bray-Curtis distance at the OTU level

High-throughput sequencing of the rhizosphere and root samples at all three growth stages and four fertilization levels was performed. The bacterial community compositions in the rhizosphere and root samples under different growth stages and N fertilization levels are shown in Fig. 2. Among the rhizosphere samples, the bacterial community composition was notably different among the four N fertilization levels at the tillering stage (Fig. 2a). The relative abundance of *Bacteroidetes* decreased, whereas that of *Actinobacteria* and *Proteobacteria (Alphaproteobacteria* and *Gammaproteobacteria,* Additional file 1: Figure S1) increased with N fertilization level. The bacterial community compositions among samples at the four N treatments in the jointing and ripening stages were more similar than those in the tillering stage. In the root samples, *Proteobacteria*, *Actinobacteria* and *Bacteroidetes* were the three dominant phyla (Fig. 2b). The relative abundance of *Actinobacteria* decreased with increasing N fertilization level, while that of *Firmicutes* was dramatically higher in the jointing stage than in the other two stages.

Principal coordinate analysis (PCoA) was performed at the operational taxonomic unit (OTU) level. The OTUs from both the rhizosphere (Fig. 2c) and the root samples (Fig. 2e) were clearly separated by plant growth stage, whereas the effect of N fertilization was observed only at the tillering stage in the rhizosphere. The dissimilarity distances among the three growth stages were calculated at each N fertilization level, and the differences in microbial community structure across the growth stages showed a decreasing trend with increasing N fertilization level in the rhizosphere (Fig. 2d). Redundancy analysis (RDA) based on microbial community structure at the OTU level showed that ROC correlated significantly with the bacterial community and accounted for 19.0% and 12.7% of the variation in the rhizosphere and root samples, respectively (Table 1). Mantel test results also revealed a significant correlation between ROC and bacterial community (Table 2).

**Fig. 3:**
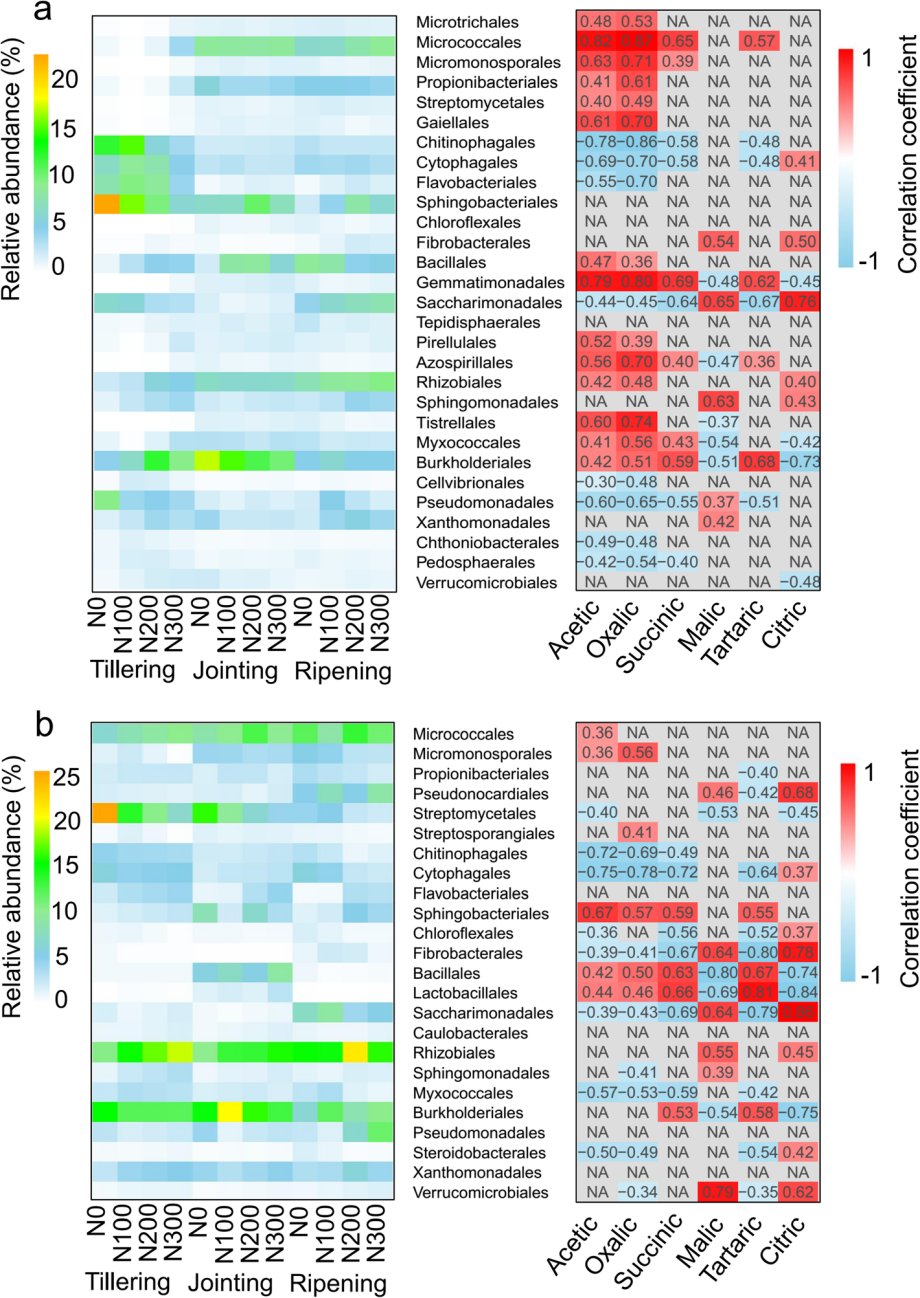
Heatmap of the dominant bacterial orders (left) and Pearson correlation analysis between the dominant bacterial orders and organic acids (right) in the rhizosphere (**a**) and root (**b**) samples. The results at the genus level are presented in the Additional file 1: Figures S4 and S5. NA indicates no significant correlation (*P* > 0.05)

Heatmaps illustrating differences in the compositions of the microbial communities among the three growth stages and at the four fertilization levels were generated for the bacterial communities at both the order (Fig. 3a, b,  Additional file 2) and genus (Additional file 1: Figures S2 and S3, Additional file 3) taxonomic levels. At the order level, the relative abundances of *Micrococcales*, *Propionibacteriales*, *Gaiellales*, *Bacillales* and *Rhizobiales* in the rhizosphere samples were significantly higher (paired t-test) at the jointing and ripening stages than at the tillering stage (Fig. 3a). In the root samples, the relative abundances of *Bacillales*, *Lactobacillales* and *Burkholderiales* were significantly greater in the jointing stage than in the other two stages. The relative abundances of *Rhizobiales* and *Sphingomonadales* at the tillering stage correlated positively with N fertilization levels, whereas that of *Streptomycetales* in the root samples showed the opposite trend across all growth stages (Fig. 3b). At the genus level (Additional file 1: Figure S2), the relative abundances of *Arthrobacter*, *Promicromonospora*, *Nocardioides*, *Streptomyces*, *Bacillus*, and *Devosia* in the rhizosphere samples were significantly higher at the jointing and ripening stages than at the tillering stage. In the root samples (Additional file 1: Figure S3), the relative abundances of *Microbacterium*, *Arthrobacter*, *Sphingomonas*, and *Devosia* correlated positively with the N fertilization level, though *Streptomyces* correlated negatively with the N fertilization level at each growth stage. In addition, the relative abundances of *Bacillus*, *Oceanobacillus* and *Lactococcus* were significantly higher in the jointing stage than in the other growth stages.

The Mantel test showed that the six organic acids correlated significantly with the root bacterial community (Table 2). To obtain an in-depth understanding of bacterial community responses to organic acids, further correlation analyses were performed between these six organic acids and bacterial orders (Fig. 3a, b) and genera (Additional file 1: Figures S2 and S3). Acetic acid, oxalic acid, succinic acid, and tartaric acid correlated positively with *Micrococcales*, *Gemmatimonadales, Azospirillales* and *Burkholderiales* in the rhizosphere samples and *Sphingobacteriales*, *Bacillales*, and *Lactobacillales* in the root samples and negatively with *Chitinophagales*, *Cytophagales*, *Saccharimonadales* and *Pseudomonadales* in the rhizosphere samples and *Cytophagales, Fibrobacterales, Saccharimonadales* and *Myxococcales* in the root samples (Fig. 3a, b). At the genus level, acetic acid, oxalic acid, succinic acid, and tartaric acid correlated positively with *Arthrobacter*, *Devosia*, *Massilia* in the rhizosphere samples and *Arthrobacter*, *Micromonospora*, *Nonomuraea*, *Pedobacter*, *Bacillus*, *Oceanobacillus*, *Lactococcus*, *Massilia* and *Stenotrophomonas* in the root samples and negatively with *Chitinophaga*, *Niastella*, *Taibaiella*, *Ohtaekwangia*, *Mucilaginibacter*, and *Acidibacter* in the rhizosphere samples and *Niastella* and *Ohtaekwangia* in the root samples (Additional file 1: Figures S2 and S3).

### Fungal community responses to plant development and N fertilization

**Fig. 4:**
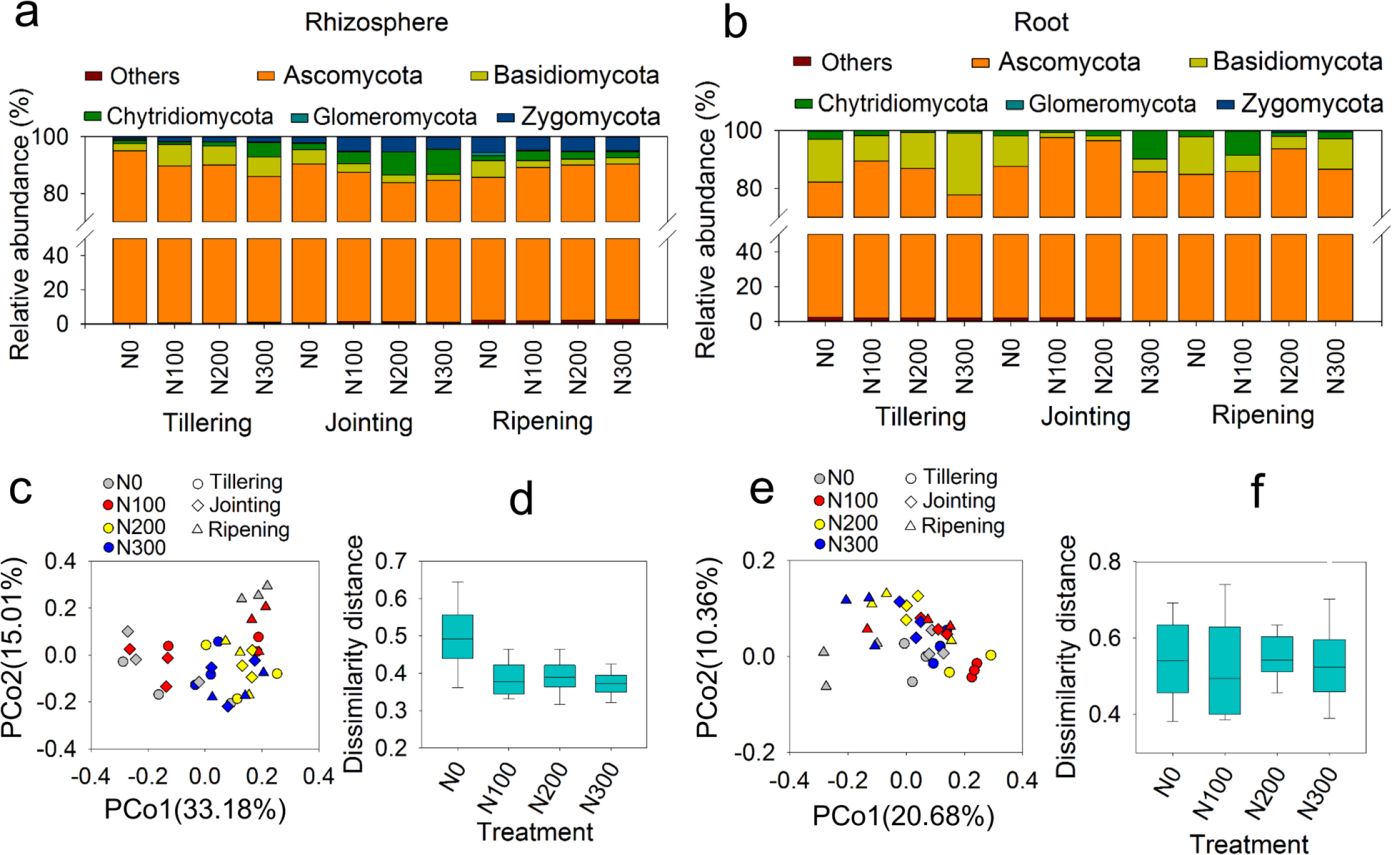
Fungal community composition of the rhizosphere (**a**) and root (**b**) samples. PCoA of the fungal communities in the rhizosphere (**c**) and root (**e**) samples. Dissimilarity distance in the rhizosphere (**d**) and root (**f**) samples. PcoA and dissimilarity distance were based on Bray-Curtis distance at the OTU level

The fungal community compositions at the phylum level in the rhizosphere and root samples are shown in Fig. 4a and b, respectively. *Ascomycota* was the dominant phylum (> 75%) in both the rhizosphere and root samples. In the rhizosphere samples, the relative abundance of *Chytridiomycota* was higher at the jointing stage than at the tillering stage, and that of *Zygomycota* was higher at the jointing and ripening stages (Fig. 4a). The dominant fungal orders (relative abundance > 1%) were *Capnodiales* (5–17%), *Pleosporales* (5–31%) and *Hypocreales* (13–30%) in the rhizosphere samples and *Capnodiales* (6–23%), *Pleosporales* (5–30%) and *Hypocreales* (20–62%) in the root samples (Additional file 1: Figure S4, Additional file 4). The identified fungal orders did not show a clear response pattern to N fertilization or plant development stages, except for *Calosphaeriales, Hypocreales* and *Sordariales*, which generally correlated positively with the N fertilization level in the rhizosphere; the relative abundance of *Pleosporales* in the root samples increased with plant growth. The community composition at the genus level showed that *Fusarium* was the dominant genus (Additional file 1: Figure S5, Additional file 5), accounting for 9–23% and 16–47% of the relative abundance in the rhizosphere and root samples, respectively. In contrast to the PCoA for bacterial communities, the PCoA for fungal communities based on the relative abundance of OTUs did not show a clear separation of the samples across growth stages in the rhizosphere and root samples (Fig. 4c and e). Nonetheless, in agreement with the observations on bacterial communities, a decreasing trend of the dissimilarity distance across the growth stages with increasing N fertilization was observed in the rhizosphere samples (Fig. 4d). The RDA showed that the SAC and SOC accounted for 12.6 and 14.8% of the variation in the rhizosphere fungal community, respectively, which were dramatically higher than that explained by ROC (Table 1), suggesting that the fungal community was strongly affected by carbon from the rhizosphere. The Mantel test showed significant correlations between the SAC and fungal community in the root samples (Table 2).

**Table 1:** Bacterial and fungal community variance explained by various carbon pools according to redundancy analysis (RDA)

Carbon pool	Explained variance			
	Bacteria		Fungi	
	Rhizosphere	Root	Rhizosphere	Root
ROC	19.0%***	12.7%***	-	-
SAC	-	7.3%*	12.6%*	11.3%*
SOC	-	7.7%*	14.8%*	-

Significance level: *P*< 0.05, *; *P*< 0.01, **; *P*< 0.001, ***. ROC: root-released organic carbon; SAC: soil active carbon; SOC: soil organic carbon.

The analysis was performed at the OTU level

**Table 2:** Correlations between bacterial and fungal communities and root organic acids and carbon pools in the rhizosphere and roots

Carbon pool	Bacteria				Fungi			
	Rhizosphere		Root		Rhizosphere		Root	
	r	*P* value	r	*P* value	r	*P* value	r	*P* value
Acetic acid	-	-	**0.174**	**0.021**	-	-	-	-
Oxalic acid	-	-	**0.228**	**0.006**	-	-	-	-
Succinic acid	-	-	**0.265**	**0.004**	-	-	-	-
Malic acid	-	-	**0.332**	**0.002**	-	-	-	-
Tartaric acid	-	-	**0.323**	**0.001**	-	-	-	-
Citric acid	-	-	**0.228**	**0.006**	-	-	-	-
ROC	**0.466**	**0.001**	**0.326**	**0.001**	-	-	-	-
SAC	**0.165**	**0.021**	**0.192**	**0.010**	-	-	**0.144**	**0.028**
SOC	-	-	**0.215**	**0.010**	-	-	-	-

The Mantel test was performed using the Pearson correlation method. Carbon pools were calculated based on the Euclidean distance, and the microbial community structures (OTU level) were calculated based on the Bray-Curtis distance. *P* is the significance level. Values with *P*<0.05 are shown. ROC: root-released organic carbon; SAC: soil active carbon; SOC: soil organic carbon

### Correlations between bacteria and fungi

**Fig. 5:**
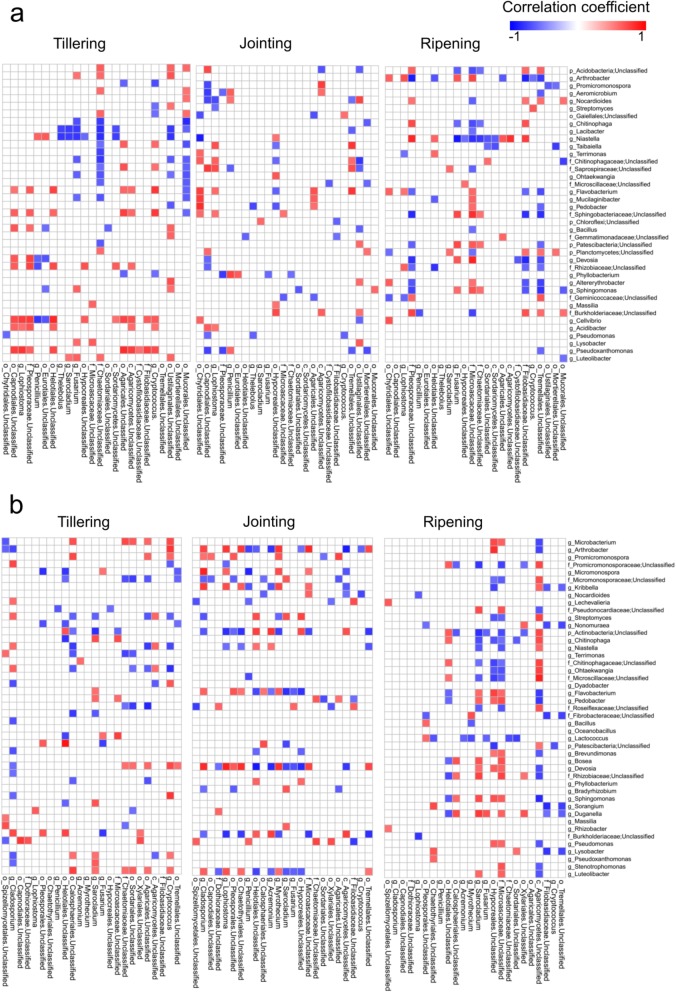
Correlation pattern of the dominant (relative abundance > 1%) bacterial and fungal genera in rhizosphere (**a**) and root (**b**) samples at three growth stages (tillering, jointing and ripening). Statistical significance was determined for all pairwise comparisons using Spearman’s method; only significant correlations, either positive (blue squares) or negative (red squares) (*p* value < 0.05), are displayed

Correlations between bacteria and fungi in the rhizosphere and root samples were assessed at the three growth stages. Genera with a relative abundance greater than 1% were used for this analysis, with 39 bacterial and 24 fungal genera in the rhizosphere samples and 45 bacterial and 24 fungal genera in the root samples. Among the bacteria and fungi in the rhizosphere samples (Table 3 and Fig. 5a), 123, 82 and 100 significant correlations (*p* < 0.05) were found at the tillering, jointing and ripening stages, respectively. *Cellvibrio*, *Niastella*, and *Pseudoxanthomonas* at the tillering stage and *Niastella* and *Arthrobacter* at the ripening stage correlated significantly with more than nine fungal genera. Among the bacteria and fungi in the root samples, 106 significant correlations were found at the tillering stage, which increased to 128 at the jointing stage and 130 at the ripening stage (Table 3). At the jointing stage, *Devosia*, *Arthrobacter* and *Luteolibacter* correlated significantly with more than nine fungal genera (Fig. 5b).

**Table 3:** Number of correlations between bacterial and fungal genera in the rhizosphere and root samples

Correlation coefficient		Number of correlations					
		Rhizosphere			Root		
		Tillering	Jointing	Ripening	Tillering	Jointing	Ripening
r≥ 0.57 (*P*<0.05)	Positive	72	41	47	52	61	59
	Negative	51	41	53	54	67	71
	Sum	123	82	100	106	128	130
r ≥ 0.65 (*P*<0.05)	Positive	27	20	21	27	45	36
	Negative	39	16	30	32	41	42
	Sum	66	36	51	59	86	78
r ≥ 0.75 (*P*<0.05)	Positive	12	7	14	7	17	7
	Negative	14	4	5	11	18	12
	Sum	26	11	19	18	35	19

## Discussion

Nitrogen fertilizer application is one of the most crucial agricultural practices and has contributed to the increase in global crop production in the last half century [32]. Previous studies have demonstrated that excessive N fertilization above a certain threshold value does not promote further crop productivity but can lead to large N losses and cause a series of environmental problems [6, 33]. The overuse of N fertilizer is currently one of the major issues in agricultural production in China [34], particularly in intensive agricultural areas such as the North China Plain (NCP), where increasing the N use efficiency and reducing the level of N fertilization remain challenging [6]. Due to the essential function of bacteria and fungi in nitrogen turnover in the root zone, a comprehensive study of the responses of root-associated microbiomes to N fertilization is necessary, especially in association with root exudation, which has been shown to be critical for plant N uptake [14].

In our study, the quantities of the soil active carbon (SAC) and soil organic carbon (SOC) in the rhizosphere under different N fertilization treatments (N100, N200 and N300) significantly increased compared with those of the control (N0). These changes can be tentatively attributed to the long-term effect of N fertilization, which increased the root biomass as well as the total amount of root exudates. In addition, because the crop residue was returned to the soil, the increased biomass of the aboveground crops due to N fertilization also contributed to the increased SAC and SOC levels in the long term. The rhizosphere SAC and SOC did not fluctuate with plant growth stages as did the root-released organic carbon (ROC) (Fig. 1), suggesting that the differences in SOC and SAC among different fertilization levels are mainly generated by cumulative changes in root exudates and crop residue return over the 20 years of cultivation.

The bacterial community structures in the rhizosphere were clearly separated by the level of N fertilization at the tillering stage but clustered together at the jointing and ripening stages (Fig. 2c). Since the ROC level was significantly higher in the jointing and ripening stages than in the tillering stage and both redundancy analysis (RDA) and the Mantel test suggested that the bacterial community is strongly related to ROC, the increased similarity among microbial communities across N fertilization levels might have been because the influence of root exudates overrode the effect of N availability in the rhizosphere.

Plant growth-promoting rhizobacteria (PGPR) are in close contact with roots and can enhance the adaptive capacity of host plants in their environments [35]. In this study, the relative abundances of *Arthrobacter*, *Bacillus*, *Massilia* and *Devosia* in the rhizosphere and *Bacillus, Oceanobacillus, Lactococcus* and *Massilia* in the roots were higher at the jointing and ripening stages than at the tillering stage (Fig. 3a), and these genera have been described as important PGPR [36–39]. Furthermore, Pearson correlation analysis showed that these taxa correlated positively with one or several organic acids (Fig. 3b). Interestingly, *Arthrobacter, Bacillus, and Devosia* also correlated positively with the level of N input. One possible explanation for these results is that the plants responded to the elevated N input by recruiting PGPR through secretion of organic acids. Indeed, recruitment of PGPR by root-secreted organic acids has been illustrated in a number of prior studies [40–42].

The composition and quantity of organic acids also changed across N fertilization levels at all three growth stages. A straightforward explanation is that the elevated N changed the physiological status of the plants. Another possible explanation for this phenomenon is that surplus N input caused the depletion of other nutrients in the soil, such as phosphate. Adjusting the quantity and composition of root exudate is a strategy developed by plants to cope with limited nutrients. In support of this notion, secretion of organic acids has been identified as an efficient way by which phosphate is released from inorganic complexes in soil [29, 43].

Fungal communities in the rhizosphere are affected by plant growth stage, soil characteristics and plant species [21, 44, 45]. A recent study also showed that priming effects caused by litter application may enhance rhizosphere activity by promoting fungal growth [46]. However, in this study, the growth stage had no significant effects on the fungal community structure (Fig. 4). In addition, RDA suggested a substantial influence of SAC on the fungal community structure in both the rhizosphere and root samples (Table 1). A recent study showed that plant endosphere fungi are a subset of fungi recruited from the surrounding soil [47], therefore, it is not surprising to find that both root and rhizosphere fungi are closely related to the edaphic factors of the surrounding soil.

## Conclusions

Both plant development and long-term N fertilization strongly influence the structure of root-associated microbiomes. In both root-associated compartments, the bacterial community composition was closely related to ROC, whereas the fungal community was associated with the rhizosphere SAC. Plant growth stage showed different effects on the correlation between bacterial and fungal communities in the root and rhizosphere samples. A number of PGPR were found to be correlated with organic acids and the N fertilization level, suggesting that the secretion of organic acids to recruit beneficial microorganisms might be an important strategy used by plants to cope with nitrogen input. This study represents a step toward a more mechanistic understanding of how shifts in microbial community composition mediate and reflect the effects of nitrogen input in intensive agricultural ecosystems.

## Methods

### Field experiment and sample collection

A long-term N fertilization field experiment was initiated in 1998 at the Luancheng Agroecosystem Experimental Station in Luancheng County, Hebei Province, China (37°53′N, 114°41′E, elevation 50 m). The experiment included four N fertilization levels, 0, 100, 200 and 300 kg N ha^−1^ per wheat-growing season, applied to triplicate plots. The soil used in this study was fluvo-aquic soil with a pH of 7.53–7.95, a total carbon (TC) of 17.03–20.80 g kg^−1^ and a total nitrogen (TN) of 1.13–1.70 g kg^−1^ [48]. Rhizosphere and root samples were collected three times during the wheat growing season in November 2016 (Feekes growth stage 2-3), March 2017 (Feekes stage 6-7) and May 2017 (Feekes stage 11), which are referred to as tillering, jointing and ripening stages in this study, respectively. Three replicate samples of root cores were collected from plants under all N fertilization levels at each growth stage. The rhizosphere samples in this study were strictly defined as the soil within 2 mm of the root surface [49]. After gently shaking the roots to remove loosely attached soil clumps, the rhizosphere samples were carefully collected by brushing the remaining soil off of the roots [50]. To decrease the impact on arbuscular mycorrhizal fungi attached to the roots and downstream DNA extraction, the roots were washed with sterilized distilled water and used for root exudate and root microbial community analyses. We therefore define the “root microbiome” in this study as the microbial communities in the root endosphere and root surface since the sample collection method did not discriminate between these two compartments [16].

### Determination of SAC, SOC, ROC and organic acids

Because only rhizosphere and root samples were investigated, soil organic carbon (SOC) and soil active carbon (SAC) in this study refer to the rhizosphere SOC and SAC. SOC is defined in the conventional way and refers to the carbon component of organic compounds in the soil. Root-released organic carbon (ROC) is defined in this study as the total carbon in the root exudate (normalized per gram of root). SAC was determined using the potassium permanganate (KMnO_4_) oxidizable C method [31, 51]. Briefly, 1.0 g of air-dried soil was mixed with 20 ml of KMnO_4_ at a concentration of 0.02 M and shaken at 200 rpm for 2 min at 25 °C. Next, the sample was centrifuged at 950×*g* for 5 min, and the supernatant was diluted with deionized water at a ratio of 1:50. The absorbance of the diluted sample at 550 nm was measured using an ultraviolet spectrophotometer (UV-2450, Shimadzu). The range of the standards was chosen to adequately cover the concentration of the samples. The change in the concentration of KMnO_4_ was used to estimate the amount of oxidized carbon, assuming that 1 mM MnO_4_^−^ is consumed (Mn (VII) to Mn (II)) during the oxidation of 0.75 mM or 9 mg of carbon. SOC was measured using the K_2_Cr_2_O_7_-H_2_SO_4_ oxidation method [52].

Root exudates were extracted by shaking 0.4 g of fresh roots with 1.5 ml of sterilized deionized water for 30 min at 1400 rpm [26, 53]. The samples were subsequently centrifuged for 5 min at 13,000×*g,* and the supernatants were filtered through a 0.22 μm syringe filter. Next, 0.5 ml of the filtered supernatants was assessed using a total organic carbon analyzer for ROC determination. Organic acids were measured using a high-performance liquid chromatograph (Waters e2695, Milford, MA, USA) equipped with a reversed-phase silica C18 column (Atlantis T3, 250 × 4.6 mm, 5 μm, Waters); 10 μl of root exudate sample was eluted with 20 mM sodium phosphate buffer (pH 2.73) at a flow rate of 0.5 ml min^− 1^ at 30 °C. Absorbance at 210 nm was monitored, and calibration curves were constructed with standard organic acids.

### DNA extraction and amplicon sequencing

Total genomic DNA was extracted from 0.5 g of rhizosphere soil or 0.4 g of fresh root powder that was obtained by grinding with liquid nitrogen using an E.Z.N.A.® Soil DNA Kit (Omega Biotek, Inc., Norcross, GA) following the manufacturer’s protocol. The 16S and 18S rRNA genes were amplified with the primer pairs 341F:785R [54] and FR1:FF390 [55], respectively. The primers contained overhanging bases to connect the Illumina sequencing adapters and dual-index barcodes in a second round of PCR. PCR was performed in a 25 μl mixture containing 12.5 μl of PCR premix (Phanta Max Super-Fidelity DNA Polymerase, Vazyme Biotech Co., Ltd., China), 1 μl of each primer (10 μM), and 1 μl of DNA template (approximately 20 ng of DNA). The PCR conditions were as follows: 95 °C for 3 min; 25 cycles of 30 s at 95 °C, 30 s at 55 °C and 30 s at 72 °C; and a final extension at 72 °C for 10 min. The PCR products were examined by agarose gel electrophoresis and then purified using AMPure XP beads (Beckman Coulter, Inc., Brea, CA) following the manufacturer’s protocol. Subsequent eight-cycle PCR was carried out to add dual-index barcodes and Illumina sequencing adapters to each sample, after which the PCR products were purified using AMPure beads. Equal molar amounts of the PCR products from each sample were mixed and sequenced using the Illumina MiSeq PE300 platform (GENEWIZ, Suzhou, China). The sequencing data were deposited in the European Nucleotide Archive under accession number PRJEB33393.

### Analysis of sequencing data

Sequences were analyzed using the Quantitative Insights Into Microbial Ecology (QIIME) pipeline [56]. The adaptor sequence, barcode and 30 low-quality bases at the end of each read were removed, after which forward and reverse reads were joined using the fastq-join method with a minimum overlap of 20 bp and a maximum mismatch within the overlap region of 10%. Low-quality sequences (Phred quality score Q < 20 or a length shorter than 200 bp) were discarded, and chimeras were filtered out using the UCHIME algorithm in the USEARCH program [57]. The high-quality data were clustered into operational taxonomic units (OTUs) at a 97% similarity using the UCLUST method [58]. The SILVA 16S and 18S rRNA databases were used as bacterial and fungal reference databases, respectively. The high-quality sequences were analyzed after removing singletons and OTUs assigned as neither bacteria nor fungi.

After completing the quality control steps, 9003–33,523 and 5811–27,012 bacterial sequences per sample were obtained from the rhizosphere and root samples, respectively. Bacterial OTU tables for the rhizosphere and root samples were subsampled to 8500 and 5500 sequences per sample, respectively. The subsampled sequences were clustered into 1002–3256 OTUs (2588 on average) for the rhizosphere samples and 817–2031 OTUs (1573 on average) for the root samples. For 18S rRNA gene sequences, 4777–29,260 and 1492–5413 high-quality sequences per sample were generated from the rhizosphere and root samples, respectively, after quality control. The fungal libraries for the rhizosphere and root samples were subsampled to 4000 and 1000 sequences per sample, respectively. The subsampled sequences were clustered into 704–1084 OTUs (895 on average) for the rhizosphere samples and 192–301 OTUs (263 on average) for the root samples. Preliminary analysis of similarities (ANOSIM) based on pooled sequences revealed significant differences (*P* < 0.001) between the rhizosphere and root samples for both the bacterial and fungal communities; therefore, sequence analyses on the rhizosphere and root samples were performed separately.

### Statistical analyses

Statistical analyses were conducted using SPSS20.0 (IBM, Chicago, USA) and R [59]. Analysis of variance and least significant difference (LSD) analysis were performed to test the significance of the effect of N fertilization level on SOC, SAC and root exudates using SPSS 20.0. Redundancy analysis and the Mantel test were performed using the vegan library in R [60] to determine correlations between carbon pools and microbial communities at the OTU level. Pearson correlation analysis between organic acids and bacterial taxa and between bacterial and fungal taxa was performed using the psych library in R [61]. ANOSIM [62] analysis using the Bray-Curtis dissimilarity matrix was performed to determine significant differences in the bacterial and fungal communities between the rhizosphere and root samples.

## Supplementary information

**Additional file 1: Figure S1.** The relative abundance of the classes within the phylum Proteobacteria in the rhizosphere and root samples under different N fertilization levels at three growth stages. **Figure S2.** Heatmap showing the relative abundance of dominant rhizosphere bacterial genera (left) and Pearson correlation analysis between dominant bacterial genera and organic acids (right). NA indicates no significant correlations (*P* > 0.05). Unclassified indicates an unidentified genus in the preceding taxa. **Figure S3.** Heatmap showing the relative abundance of dominant bacterial genera in the root samples (left) and Pearson correlation analysis between dominant bacteria genera and organic acids (right). NA indicates no significant correlations (*P* > 0.05). Unclassified indicates an unidentified genus in the preceding taxa. **Figure S4.** Heatmap showing the relative abundance of dominant fungal orders in the rhizosphere and root samples under four fertilization levels at three growth stages. **Figure S5.** Heatmap showing the relative abundance of dominant fungal genera in the rhizosphere and root samples under four fertilization levels at three growth stages. Unclassified indicates an unidentified genus in the preceding taxa.

**Additional file 2.** Dominant bacterial genera (relative abundance > 1%) in the rhizosphere and root samples.

**Additional file 3.** Dominant bacterial genera (relative abundance > 1%) in the rhizosphere and root samples.

**Additional file 4.** Dominant fungal orders (relative abundance > 1%) in the rhizosphere and root samples.

**Additional file 5.** Dominant fungal genera (relative abundance > 1%) in the rhizosphere and root samples.

## Data Availability

All sequencing data used in this study are available in the European Nucleotide Archive under accession number PRJEB33393.

## Abbreviations

PCoA: Principal coordinate analysis
PGPR: Plant growth-promoting rhizobacteria
RDA: Redundancy analysis
ROC: Root-released organic carbon
SAC: Soil active carbon
SOC: Soil organic carbon
TC: Total carbon
TN: Total nitrogen
