# Supplementary material for: Root-associated microbiomes of wheat under the combined effect of plant development and nitrogen fertilization
Source: Microbiome. 2019 Oct 22;7:136. doi: 10.1186/s40168-019-0750-2 (PMC6806522; doi:10.1186/s40168-019-0750-2)
Supplement: Supplementary file 1 — Additional file 1: Figure S1. The relative abundance of the classes within the phylum Proteobacteria in the rhizosphere and root samples under different N fertilization levels at three growth stages. Figure S2. Heatmap showing the relative abundance of dominant rhizosphere bacterial genera (left) and Pearson correlation analysis between dominant bacterial genera and organic acids (right). NA indicates no significant correlations (P > 0.05). Unclassified indicates an unidentified genus in the preceding taxa. Figure S3. Heatmap showing the relative abundance of dominant bacterial genera in the root samples (left) and Pearson correlation analysis between dominant bacteria genera and organic acids (right). NA indicates no significant correlations (P > 0.05). Unclassified indicates an unidentified genus in the preceding taxa. Figure S4. Heatmap showing the relative abundance of dominant fungal orders in the rhizosphere and root samples under four fertilization levels at three growth stages. Figure S5. Heatmap showing the relative abundance of dominant fungal genera in the rhizosphere and root samples under four fertilization levels at three growth stages. Unclassified indicates an unidentified genus in the preceding taxa. [file 40168_2019_750_MOESM1_ESM.docx]

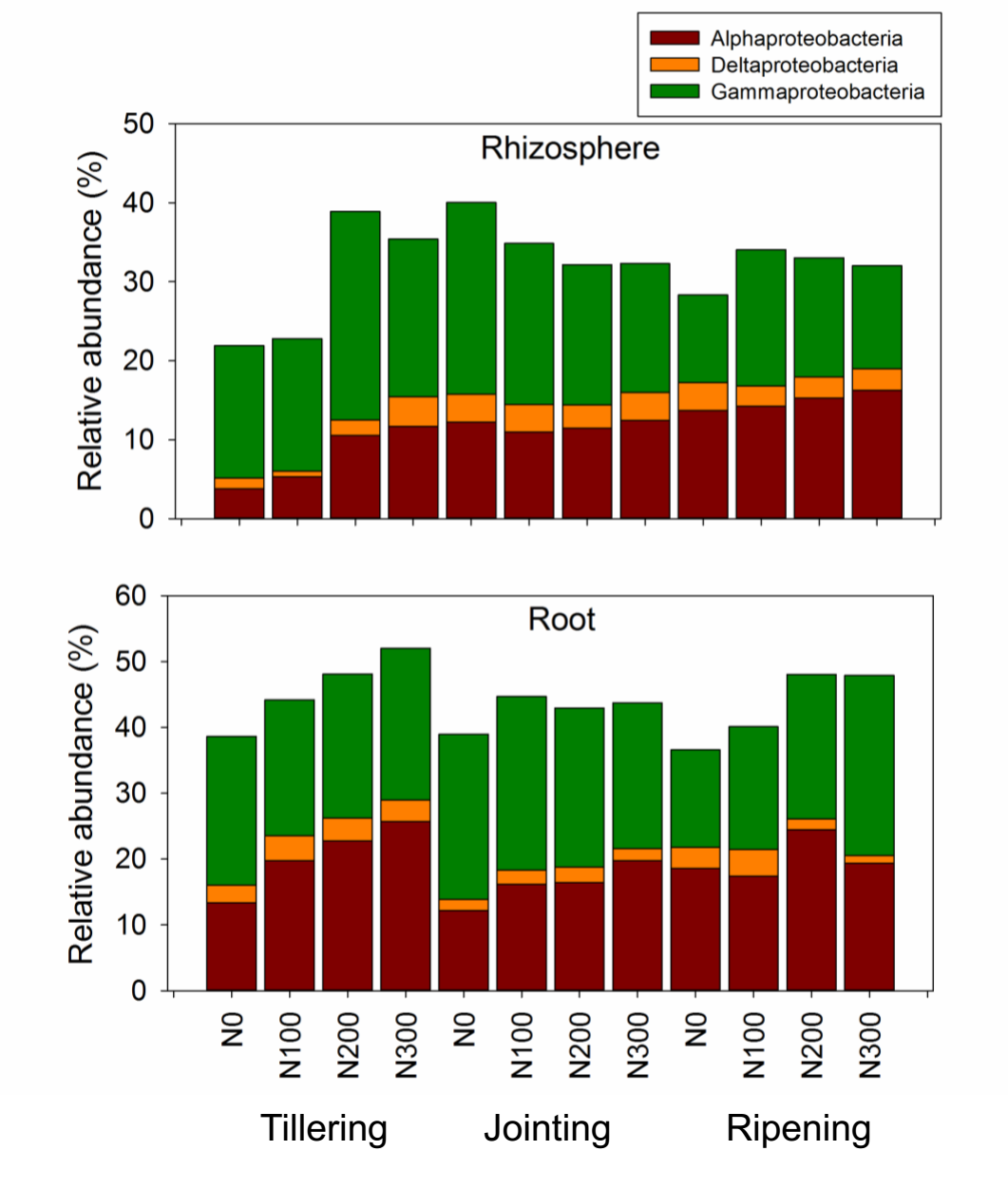


Fig. S1. The relative abundance of the classes within the phylum Proteobacteria in the rhizosphere and root samples under different N fertilization levels at three growth stages.


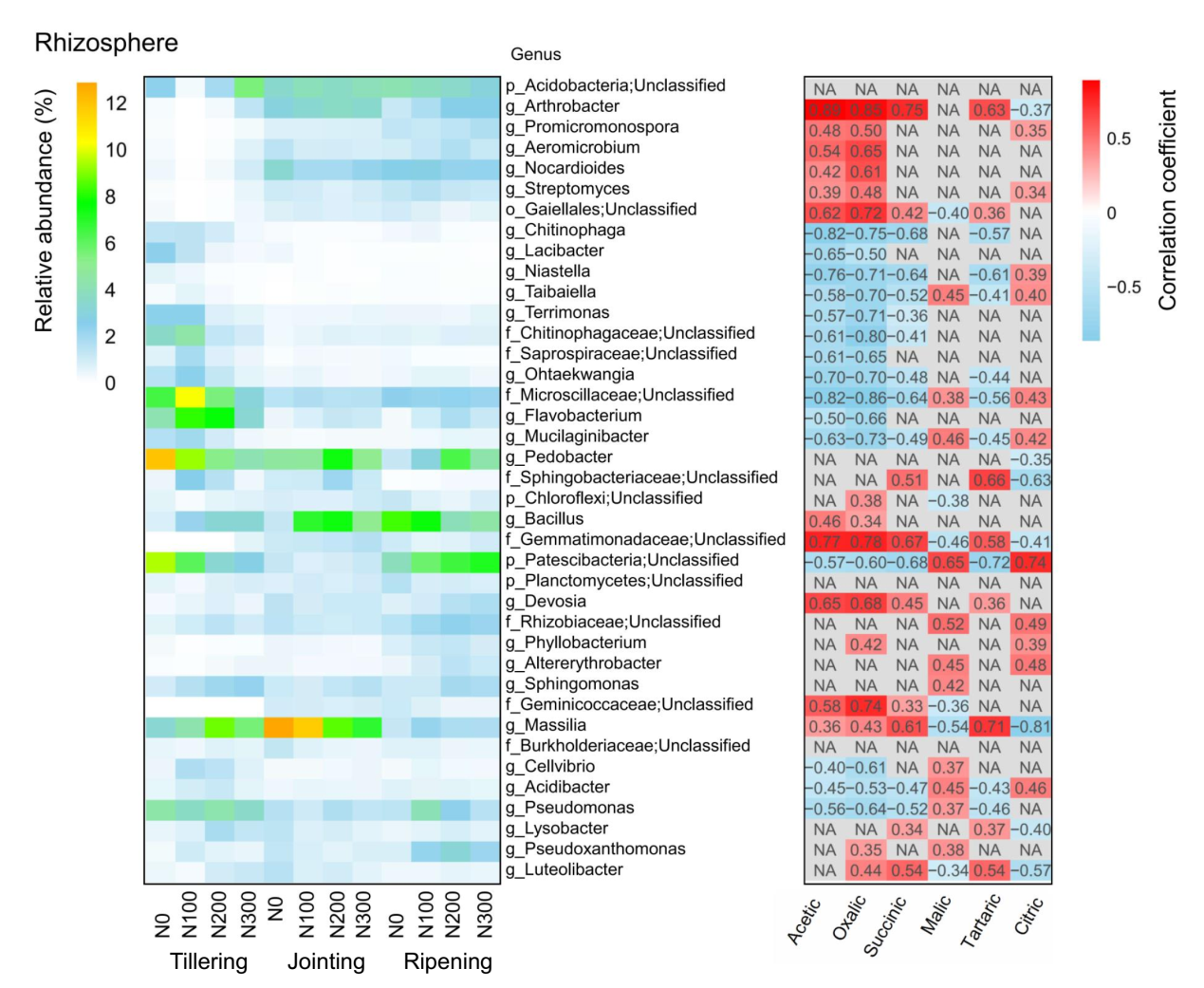


Fig. S2. Heatmap showing the relative abundance of dominant rhizosphere bacterial genera (left) and Pearson correlation analysis between dominant bacterial genera and organic acids (right). NA indicates no significant correlations (*P*>0.05). Unclassified indicates an unidentified genus in the preceding taxa.


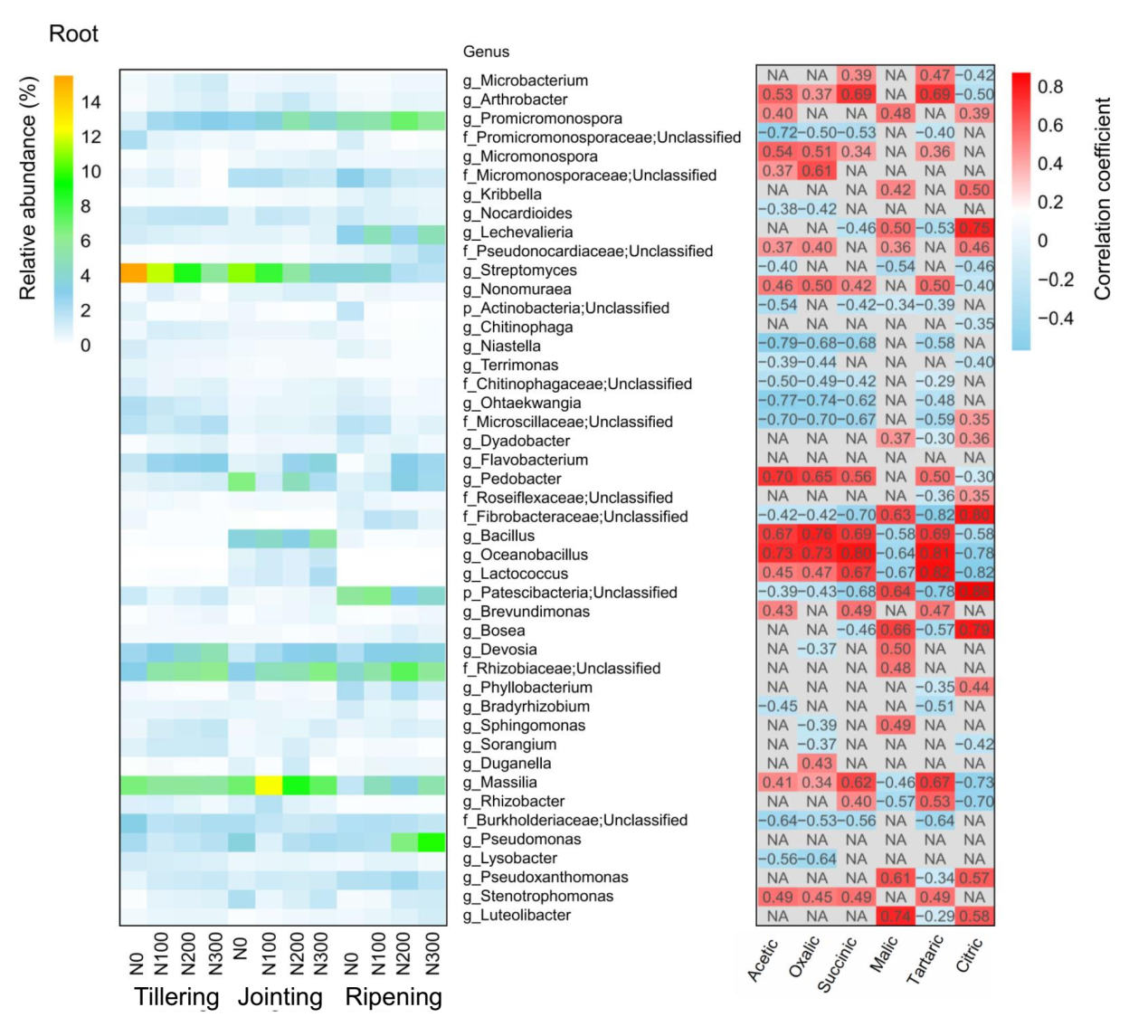


Fig. S3. Heatmap showing the relative abundance of dominant bacterial genera in the root samples (left) and Pearson correlation analysis between dominant bacterial genera and organic acids (right). NA indicates no significant correlations (*P*>0.05). Unclassified indicates an unidentified genus in the preceding taxa.


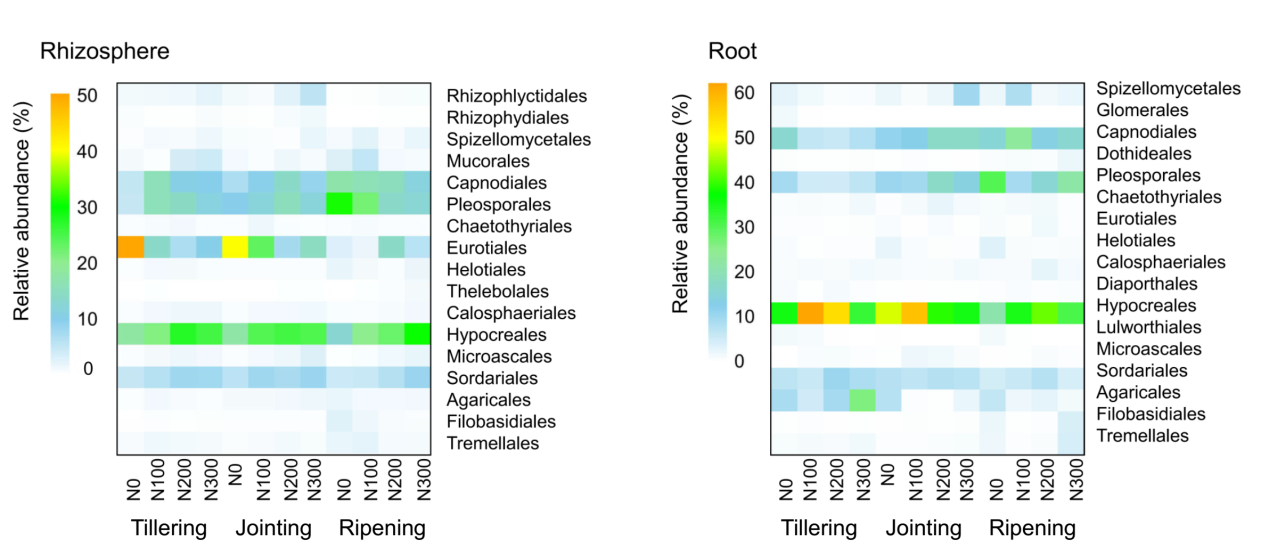


Fig. S4. Heatmap showing the relative abundance of dominant fungal orders in the rhizosphere and root samples under four fertilization levels at three growth stages.


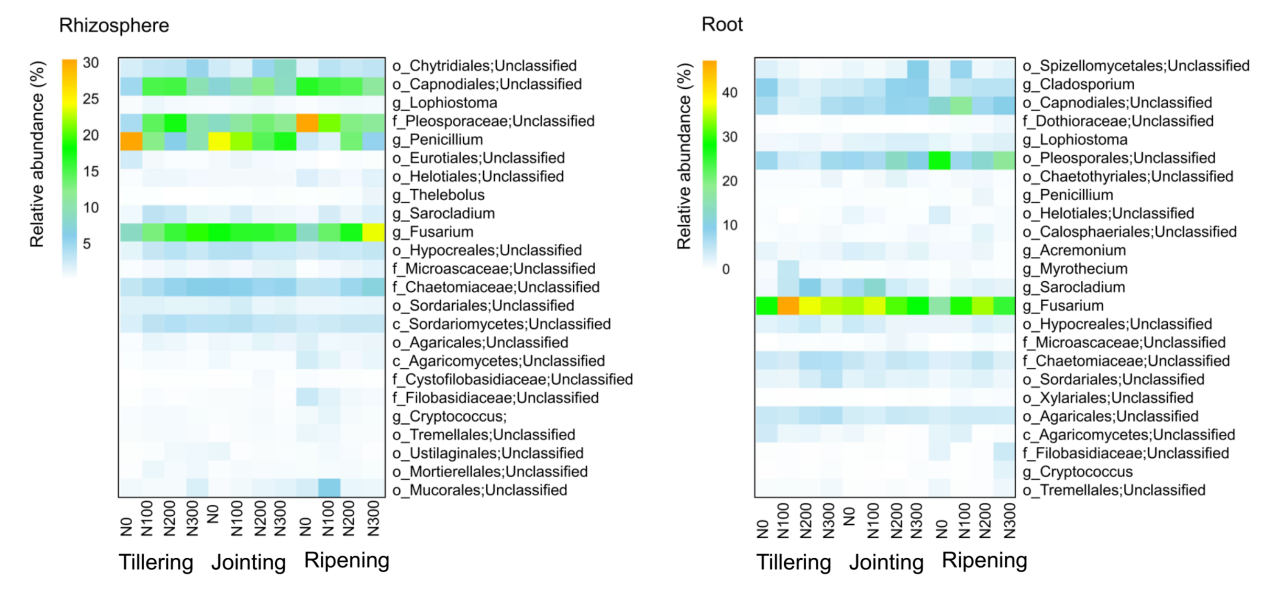


Fig. S5. Heatmap showing the relative abundance of dominant fungal genera in the rhizosphere and root samples under four fertilization levels at three growth stages. Unclassified indicates an unidentified genus in the preceding taxa.
